# Impact of Juice Extraction Method (Flash Détente vs. Conventional Must Heating) and Chemical Treatments on Color Stability of Rubired Juice Concentrates under Accelerated Aging Conditions

**DOI:** 10.3390/foods9091270

**Published:** 2020-09-10

**Authors:** Richard G. Ntuli, Ravi Ponangi, David W. Jeffery, Kerry L. Wilkinson

**Affiliations:** 1 E & J Gallo Winery, Process Technology, P.O. Box 1130, Modesto, CA 95353, USA; richard.ntuli@ejgallo.com (R.G.N.); ravi.ponangi@ejgallo.com (R.P.); 2 School of Agriculture, Food and Wine, The University of Adelaide, PMB 1, Glen Osmond SA 5064, Australia; kerry.wilkinson@adelaide.edu.au

**Keywords:** grape color extraction, grape food coloring, color units, color degradation kinetics, grape concentrate

## Abstract

Low color stability of Rubired food and beverage coloring negatively impacts color yield during production and storage while also limiting the use of this type of food colorant in applications where color stability is a key requirement. This study investigated the impact on color stability of using flash détente (FD) for Rubired color extraction in comparison to a conventional must heating (CMH) extraction process, in conjunction with the use of commercial seed tannin, acetaldehyde, or acid to lower the pH. Rubired concentrate color was evaluated under accelerated aging conditions at 50, 60, and 70 °C, over zero, three, six, and nine days for the different treatments. FD concentrate had lower color stability, with a half-life of 203.3 h and activation energy of 59.2 kJ/mol at 50 °C compared to the CMH concentrate with 233.9 h and 65.2 kJ/mol. FD concentrate generated less 5-hydroxymethylfurfural (5-HMF) during accelerated aging regardless of treatment. Acetaldehyde, low pH, and the combination of these two treatments increased red color stability as well as violet and brown color, whereas seed tannin had no effect. Low pH treatments increased 5-HMF formation and browning, which was detrimental to concentrate quality. Although promising in terms of color stabilization, implementation of these treatments will require development of solutions to mitigate the production of 5-HMF.

## 1. Introduction

Color stability is an important quality attribute for Rubired grape concentrate, considering its main application as a beverage or food colorant. This is especially important in view of the increased consumer interest in the use of natural food colorants due to their health benefits [1], in place of synthetic counterparts that have been reported to adversely affect human health [2]. The biggest challenge in replacing synthetic dyes with natural ones is matching the vivid colors and high color stability of the former. Enhanced red color stability during processing and storage of Rubired concentrate could potentially increase the range of food and beverage applications for the concentrate and increase its attractiveness as a replacement for synthetic food dyes.

Red color stability in processed red grape juice or concentrate is influenced by grape variables, as well as production and storage conditions. Production conditions that reportedly influence red grape concentrate color stability include extraction temperature and duration [3]. These factors impact the stability of anthocyanins by affecting enzymatic activity of polyphenol oxidase, peroxidases, and other oxidative enzymes, as well as β-glucosidase enzymes that can accelerate anthocyanin degradation [4]. Increasing juice extraction temperature from 60 to 80 °C has been reported to increase acidity, as well as the concentrations of total anthocyanins and phenolic compounds, whereas at higher temperatures, i.e., around 90 °C, acidity, anthocyanins, and phenolic compounds decreased [5]. Increased acidity and extraction of phenolic compounds would be expected to increase color stability, as anthocyanins are more stable at lower pH values (i.e., at pH < 3) [6], and various phenolic compounds are known to act as color stabilizing co-pigmentation cofactors, whereas others react with anthocyanins to form more stable polymeric pigments [7].

In terms of extraction technologies, flash détente (FD) employs a high extraction temperature of ~85 °C, compared to ~60 °C for conventional must heating (CMH) extraction [8]. The former involves a relatively short duration of heating followed by rapid vacuum cooling of must. In contrast, the CMH extraction process typically has longer extraction times, ranging from 30 to 120 min, and then very slow, natural cooling of must and juice, prior to evaporation into concentrate. Based on these different heating conditions, it could be expected that concentrates produced via the two processes would have different color stabilities.

Degradation of juice or concentrate color during storage is impacted by several factors, including extraction temperature [3], contact time, grape variety, maturity at harvest [3], pH, light, the presence of antioxidants, pro-oxidants, oxidative enzymes [4,6,9,10], sodium chloride and transition metal ions [11,12,13], flavanols and other grape derived compounds, and storage temperature [3,14,15]. Of the factors that have been shown to influence red color stability, only antioxidant addition and cold temperature storage are regularly used to control color degradation during processing and storage. The most common antioxidant used in juice and wine processing is sulfur dioxide, although ascorbic acid is occasionally used. However, these two antioxidants are not effective in juice concentrate due to their tendency to bind with sugars or act as a pro-oxidant, respectively [13]. The only effective, widely used color preservation method is therefore cold storage of juice, which is a costly option, but one still practiced by commercial producers as a practical way to achieve stability. The need to develop more effective ways to stabilize red juice or concentrate color is therefore an obvious one.

Approaches that show promise for Rubired concentrate color stabilization, based on findings in other fruit juices, concentrates and wines, include the use of co-pigmentation cofactors [16,17,18,19,20,21], pH adjustment, and reaction of phenolic and non-phenolic compounds with anthocyanins to form more stable pigments, such as polymeric pigments and pyranoanthocyanins [22,23]. Because of consumer demand for minimally processed foods and food additives derived from natural food materials [24], priority should be given to treatments utilizing grape or fermentation-derived cofactors, and phenolic and non-phenolic compounds that can stabilize anthocyanins. However, more research is needed to screen these grape and wine constituents to determine which are the most effective, and to develop practical methods for stabilizing red concentrate color.

The goal of this work was to compare and contrast the red color stability and degradation kinetics of Rubired concentrate derived from FD with that from CMH. To date, no study has been documented that compares the red color stability, brown and violet color change, and formation of 5-hydroxymethylfurfural (5-HMF) in FD-derived red grape concentrate to that of traditionally extracted concentrate. The authors acknowledge that there are variations of the FD process in commercial application and this study was an evaluation of concentrate generated by a generic FD process rather than an evaluation of FD equipment itself. The study also aimed to investigate the effect of low pH, acetaldehyde (an alcoholic fermentation-derived additive), and grape-derived seed tannin addition on red color stability and brown and violet color evolution (using color measurements employed by commercial producers and end-users), as well as their effect on compounds that are thought to influence color expression under accelerated aging conditions.

## 2. Materials and Methods

### 2.1. Chemicals

Chemicals (analytical grade) were purchased from Sigma Aldrich (St Louis, MO, USA). Standards were sourced from Sigma Aldrich and Indofine chemicals (Hillsborough, NJ, USA). Solvents (HPLC grade) were sourced from BDH (Radnor, PA, USA). Deuterated internal standards were sourced from C/D/N Isotopes Inc. (Pointe-Claire, QC, Canada).

### 2.2. Preparation of Rubired Juice Concentrate

The Rubired juice concentrate used for this study was produced via commercial scale CMH and FD processes (Figure S1), with grapes (approximately 400 metric ton) harvested at commercial maturity (23–24 °Brix) from a vineyard located in the Central Valley region of California (36.57° N, 119.61° W), during the 2016 vintage. For CMH extraction, 180 metric tons of grapes were destemmed and crushed, and 50 mg/L sulfur dioxide (as an 8% solution of potassium metabisulfite) and pectinase (Rohavin MX, AB Enzymes, Darmstadt, Germany, 28 mL/metric ton) were added. Processing at 127 metric tons/hour, the must was heated to 57 °C using a steam-heated shell and tube heat exchanger (Wiegmann and Rose, Oakland, CA, USA). Following CMH, hot must was held for 2 h before pressing with a Diemme screw press (Diemme Enologia, Lugo, Italy). Solids were removed from the resulting juice using a Westfalia decanter centrifuge (Westfalia, Northvale, NJ, USA), prior to evaporation with a high temperature (HT) APV plate and frame evaporator (SPX Flow, Crawley, UK) to give a 55–56 °Brix concentrate. For FD extraction, 180 metric tons of grapes were destemmed and crushed, and the resulting must heated to 85 °C (for 5–10 min) using a Della Toffola flash détente unit (Della Tofolla, Trevignano, Italy) at 27 metric tons/h. Vacuum pressure in the flash chamber was maintained at -0.94 bar. Following FD, 50 mg/L of sulfur dioxide and pectinase (28 mL/metric ton) were added to the must before pressing; additions were made post-FD to avoid their loss due to vacuum flashing and inactivation, respectively, during FD processing. The resulting juice was chilled (to 4 °C to prevent fermentation) and solids were removed prior to evaporation to give 55–56 °Brix concentrate (as for CMH). FD and CMH concentrates were stored at 2–4 °C to prevent fermentation and minimize red color loss and browning.

### 2.3. Accelerated Color Stability Testing under Different Treatment Conditions

A laboratory scale accelerated aging experiment was conducted to compare the color stability of CMH and FD processes, and to investigate the impact of various chemical treatments on the color stability of Rubired concentrates produced by the two thermal processes. Treatments (performed in duplicate) comprised a control (i.e., no additions) and additions of seed tannin, acetaldehyde, acid, and combinations of these additions, as outlined below, to both CMH and FD concentrate prior to heat treatment.

#### 2.3.1. Commercial Grape Seed Tannin

A commercial grape seed tannin powder (10 g) was dissolved in 60% aqueous ethanol solution (15 mL) and added to concentrate to give 1000 mg/L gallic acid equivalents (GAE) of tannin, as measured by the Folin-Ciocalteu method [22]. The concentrate was thoroughly mixed after tannin addition.

#### 2.3.2. Acetaldehyde

Acetaldehyde in ethanol solution (50% *w*/*w*, Penta International Corp., Livingston, NJ, USA) was added to concentrate (at 4 °C, to prevent acetaldehyde from flashing off) to give a concentration of 300 mg/L. The concentrate was then thoroughly mixed.

#### 2.3.3. Acid

Concentrated hydrochloric acid (37% *w*/*v*, EMD, Burlington, MA, USA) was added to concentrate to adjust the pH to 2.8, the lowest pH permitted by Alcohol and Tobacco, Tax and Trade Bureau (TTB) regulations [25], although hydrochloric acid was used for experimental purposes only. The concentrate was thoroughly mixed during pH adjustment.

#### 2.3.4. Acetaldehyde and Acid

Concentrated hydrochloric acid was added to concentrate to adjust the pH to 2.8 before acetaldehyde was added to achieve a concentration of 300 mg/L.

#### 2.3.5. Seed Tannin, Acetaldehyde and Acid

Concentrated hydrochloric acid was added to concentrate to adjust the pH to 2.8 before acetaldehyde and seed tannin were added (as above) to give an acetaldehyde concentration of 300 mg/L and a seed tannin concentration of 1000 mg/L GAE of tannin.

#### 2.3.6. Heat Treatments

Control and treated FD- and CMH-derived concentrates (1.3 L, minus two 50 mL aliquots, as the time zero samples) were transferred into 6 × 200 mL amber colored glass bottles with black plastic lids. The bottles were filled with concentrate to leave minimal headspace and sealed to prevent moisture loss during heat treatment. Two bottles were heated in each of three ovens held at 50, 60, and 70 °C to cause accelerated aging. After three, six, and nine days of heating, samples were thoroughly mixed before aliquots (50 mL from each bottle) were collected for determination of red, brown, and violet color, total tannin, anthocyanins, monomeric flavan-3-ols, polymeric flavan-3-ols (proanthocyanidins), hydroxycinnamic acids, flavonols, and 5-HMF.

#### 2.3.7. Color Degradation Kinetics

Changes in color and phenolic data were used to determine the kinetics of red color loss, brown color evolution, and phenolic transformation in FD and CMH concentrates under accelerated aging conditions (i.e., at elevated temperatures of 50, 60, and 70 °C over nine days). The concentration, natural logarithmic concentration, inverse of concentration, and inverse of squared concentration of red, brown, and violet color and other phenolic compounds were plotted against storage time to determine reaction orders, rate constants (k), half-life, and Q_10_ values to estimate temperature dependency. Linear regression was used to determine reaction orders and Arrhenius plots (log k vs. 1/T K) were used to determine activation energies (E_a_) for the two concentrate types.

### 2.4. Compositional Analysis of Rubired Juice Concentrate

Concentrate samples were centrifuged (4000× *g* for 15 min; Beckman Coulter, Brea, CA, USA) prior to color and compositional analysis.

#### 2.4.1. Color Analysis

Color measurements were performed using a United States Department of Agriculture (USDA) spectrophotometric method for red juice concentrate [26] that specifies measuring absorbance after adjusting pH to 3.2 with McIlvaine buffer. An 8453 UV-Vis spectrophotometer (Agilent Technologies, Palo Alto, CA, USA) with sipper and 1 cm flow through cuvette were used with ChemStation control software (version B.02.01). Centrifuged juice or concentrate samples (~1 g) were accurately weighed and diluted with Mcllvaine buffer (pH 3.2) to a total volume of 100 mL. The pH-adjusted samples were thoroughly mixed before being filtered through 1 μm glass fiber filters (Pall, New York, NY, USA). This sample weight was chosen to obtain an absorbance at 520 nm in the range of 0.3–0.7 absorbance units. Absorbances were measured at 520, 430, and 580 nm for red, brown, and violet color measurement, respectively.

Color units (CU) were calculated as follows:

CU_(wavelength)_ = (Absorbance × 2000)/[(sample weight (g)) × (dilution factor)] (1)

Color units were normalized to 68 °Brix to compare color and compositional data in concentrates of different °Brix values.

Brown and violet indices were calculated as follows:

Brown index = CU_430nm_/CU_520nm_; Violet index = CU_580nm_/CU_520nm_ (2)

Because the color unit scale was based on measuring the absorbance of 2 g of sample made up to 100 mL with buffer [26] (rather than 1 g in the present case), the absorbance values were multiplied by 2 and divided by sample weight to normalize absorbance readings to 2 g of sample. The normalized absorbance was then multiplied by 1000 to convert from a decimal absorbance reading to a color units scale with whole numbers, which are easier to evaluate.

#### 2.4.2. Phenolic Analysis 

The total phenolics in concentrate samples and seed tannin additive were measured using the Folin-Ciocalteu method [27], with results reported as mg/L GAE.

Phenolics profiling of Rubired concentrate samples was undertaken (in duplicate) via HPLC analysis. Samples (25 mL) were diluted with distilled water that had been acidified to a pH of 2.0 with 1 M hydrochloric acid, to a total volume of 200 mL. Samples were then analyzed by reversed-phase chromatography using an Agilent 1200 HPLC (Agilent Technologies, Santa Clara, CA, USA) equipped with a quaternary pump, a diode array detector (DAD), and a Varian PLRP-S column (250 × 4.6 mm, 5 μm particle size, Varian Inc., Palo Alto, CA, USA) fitted with a PLRP-S guard cartridge. A binary solvent gradient was used consisting of water with 0.5% (*v*/*v*) orthophosphoric acid (85% *w*/*v*, mobile phase A), and acetonitrile with 0.5% (*v*/*v*) orthophosphoric acid (85% *w*/*v*, mobile phase B). The column thermostat was set at 50 °C and the injection volume was 20 μL.

Individual phenolic compounds were measured at the following wavelengths: proanthocyanidins at 230 nm; gallic acid, catechin, and epicatechin at 280 nm; grape reaction product (GRP), caftaric acid, and caffeic acid at 320 nm; quercetin glycosides (total of quercetin glucoside and quercetin glucuronide) and quercetin at 360 nm; and malvidin-3-*O*-glucoside, malvidin-3,5-*O*-diglucoside, and pigmented polymers at 520 nm. Standard solutions of these compounds were used for identification and quantification. Due to unavailability of pure standards for pigmented polymers and proanthocyanidins, malvidin-3-*O*-glucoside and catechin standards, respectively, were used. Pigmented polymers were reported as malvidin-3-*O*-glucoside equivalents, whereas proanthocyanidins were reported as catechin equivalents.

#### 2.4.3. 5-Hydroxymethylfurfural Analysis

Concentrate samples were diluted, extracted, purified, and analyzed (in duplicate) using a previously described method [28]. The 5-HMF concentrations of the resulting extracts were determined using an Agilent 6890 gas chromatograph coupled to an Agilent 5973 mass spectrometer (Agilent Technologies, Santa Clara, CA, USA).

### 2.5. Sediment Quantification and Testing

Heating the CMH and FD concentrates resulted in sediment formation. To measure the amount of sediment, duplicate concentrates heated at 70 °C for 12 days were thoroughly mixed and then centrifuged (in pre-weighed centrifuge tubes) at 4000 × *g* for 15 min using a swinging bucket centrifuge (Beckman Coulter, CA, USA). The resulting supernatant was decanted, and centrifuge tubes were re-weighed to determine the weight of solids. The solubility of sediments in water and 30% and 50% ethanol was also determined. All of the sediment dissolved in 30% ethanol and was subsequently analyzed for phenolic compounds (as above).

### 2.6. Statistical Analysis

Data were analyzed by one-way analysis of variance (ANOVA) and repeated measures ANOVA using Minitab (State College, PA, USA). Tukey-HSD was used for mean comparisons of the treatments. The level of significance was set at α = 0.05.

## 3. Results and Discussion

### 3.1. Red Color Stability

All color and phenolic compound composition data (with the exception of color ratios and sediment data) from the accelerated color stability trials were normalized to 68 °Brix to account for differences in the initial °Brix values of CMH and FD concentrates. Color stability testing showed that the CMH concentrate had greater red color stability compared to the FD concentrate (Table 1). After nine days at elevated temperature, FD concentrate had lost 3.0% to 4.8% more of its original color, depending on temperature.

**Figure 1:**
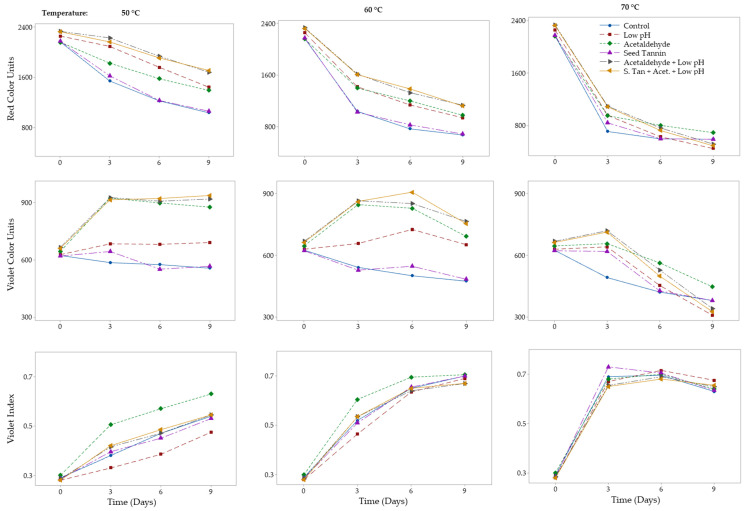
Effect of seed tannin, low pH, and acetaldehyde, individually or in combinations, on red and violet color stability (top and middle rows) and violet color index (bottom row) of Rubired concentrate derived from flash détente, heated at 50 °C (left column), 60 °C (middle column), and 70 °C (right column).

Color and phenolic compound composition data (with the exception of color ratios) from the accelerated color stability trials were normalized by calculating the impact of three, six, and nine days of heat exposure as the percentage change from their initial concentrations, to account for differences in the initial composition of CMH and FD concentrates. Repeated measures ANOVA of color data normalized to 68 °Brix and relative to the initial concentrate composition confirmed that CMH concentrate retained significantly more red color after nine days of accelerated aging at 50 °C (Table 2). The CMH concentrate had a 20% higher concentration of the more stable pigmented polymers (114 vs. 95 mg/L at 68 °Brix). Treating FD concentrate with acetaldehyde, lowering the pH, and combining these two treatments increased red color stability at 50 and 60 °C, but not at 70 °C; whereas the addition of seed tannin, either alone or in combination with other treatments, had no significant effect on color stability, regardless of temperature (Figure 1, Table 3). The ineffective red color stabilization role observed for seed tannin in concentrate was consistent with previous studies on wine [29,30], but contrary to another [31] that suggested a beneficial effect of enological tannin addition on color stability. Similar trends were seen for CMH concentrate under the same treatment conditions and temperatures, as demonstrated by the lack of significant interactions between concentrate type and treatment (Table 2).

Lowering the pH or adding acetaldehyde stabilized the red color of concentrates stored at 50 or 60 °C, and combining these treatments gave even greater stabilization due to the increased formation of pigmented polymers (Table 4) and pyranoanthocyanins such as vitisins [32,33]. After nine days at 60 °C, concentrate treated with acid or acetaldehyde had on average 35% to 38% more red color, whereas concentrate from the combined treatment had on average 62% more red color compared to untreated concentrate (Table 3).

At 70 °C, only acetaldehyde was effective at stabilizing red color. In contrast, all pH-adjusted concentrates had significantly lower red color units than their corresponding control, regardless of other treatments. The result at 70 °C under low pH conditions was in contrast to the outcomes at 50 or 60 °C and most likely due to acid hydrolysis of red pigments, which is favored at higher temperature and longer treatment duration [34]. This seemingly reversed the beneficial effect of low pH on color stability that was initially seen through increased pigmented polymer formation up to day six (data not shown). As such, anthocyanins were likely to have hydrolyzed into their less stable anthocyanidins [35], which degraded at a faster rate and resulted in less red color compared to the control.

### 3.2. Violet Color Stability

Violet color units decreased in control FD and CMH concentrates after nine days of accelerated aging, with the extent of color loss increasing with increased temperature (Table 1). Repeated measures ANOVA showed that the overall violet color units for all treatments combined (i.e., low pH, and acetaldehyde and seed tannin additions) and control significantly increased with accelerated aging (i.e., relative to time zero) to a greater extent in FD concentrate compared to CMH concentrate (Table 2). Seed tannin had no effect on violet color formation or stability, regardless of temperature (Table 3). Tannin-only treatments and controls exhibited violet color loss at all temperatures, whereas low pH, acetaldehyde, and combinations of these treatments showed violet color increases at 50 and 60 °C, and only exhibited color loss at 70 °C (Table 3). Unlike at 50 and 60 °C, where violet color markedly increased during the first three days of aging for combined low pH and acetaldehyde treatments, less of an increase was seen at 70 °C, likely due to the rapid loss of anthocyanins at the higher temperature (Figure 1).

In a similar pattern to red color, low pH, acetaldehyde addition, and a combination of these treatments gave 24–38%, 32–57% and 50–66% higher violet color units, respectively, compared to the control after nine days of accelerated aging at 50 or 60 °C (Table 3). Likewise, violet color units decreased 10–27% compared to the control for all three low pH treatments but increased 12–18% in acetaldehyde-only treatments after nine days of heating at 70 °C for both CMH and FD concentrates (Table 3). Acetaldehyde reacts with anthocyanins to form the more stable pyranoanthocyanin vitisin B [36,37], which has been reported to have greater stability [38] and contributes 11–14 times more color compared to unmodified anthocyanins due to vitisins having higher extinction coefficients [32]. Pyranoanthocyanin formation arising from reaction with acetaldehyde likely resulted in greater violet and red color expression due to a bathochromic shift in λ_max_, and the relatively high concentrate pH of ~4.0 would have favored pyranoathocyanin color expression over that of anthocyanins.

CMH concentrates had significantly higher violet to red color ratio (violet index) compared to FD concentrates after accelerated aging (Table 2). The violet index increased for all treatments involving acetaldehyde and/or low pH due to the combination of red color loss and violet color formation (Figure 1). For control and seed tannin concentrates, the rate of violet color loss was slower than for red color loss, thereby increasing the violet index. At 70 °C, the violet index plateaued for all treatments after three days of heating (Figure 1). Thereafter, the violet index remained fairly constant, potentially indicating a similar rate of violet and red color degradation. After three days of heating at 70 °C, most of the anthocyanins present in the concentrate had either been converted into non-red compounds or more stable pigments such as pigmented polymers (Table 4) and pyranoanthocyanins. This slowed red color degradation to that of violet color loss, resulting in a fairly constant violet index from three to nine days of aging (Figure 1). Red color loss from untreated concentrate was much higher than violet color loss after nine days of accelerated aging (Table 1). As such, using low pH, the addition of acetaldehyde, or a combination of these treatments to promote conversion of anthocyanins to more stable pigmented polymers and pyranoanthocyanins may provide a strategy for improving the color stability of Rubired concentrate.

### 3.3. Anthocyanins and Pigmented Polymers

Loss of malvidin-3,5-*O*-diglucoside, the most abundant anthocyanin in Rubired juice and concentrate [39], was significantly lower in FD concentrate compared to CMH concentrate (Table 2). The concentrate type by treatment interaction was not significant, suggesting the chemical treatments impacted the two concentrate types in a similar manner. There was no significant difference between malvidin-3-*O*-glucoside degradation in CMH and FD concentrates.

Concentrate storage temperature and acetaldehyde or acid treatment affected anthocyanin loss. After nine days of heating at 50, 60, and 70 °C, low pH and acetaldehyde-treated concentrates had similarly lower malvidin-3,5-*O*-diglucoside concentrations compared to the control, likely due to a faster rate of conversion of anthocyanins into pyranoanthocyanins and polymeric pigments [32]. As noted above, seed tannin had no effect on color parameters (Table 3). Pigmented polymer concentrations showed an opposing trend to anthocyanins, with acetaldehyde treatment giving significantly higher concentrations after nine days of accelerated aging at all three temperatures, compared to the low pH and seed tannin treatments and the control (Table 4).

Unlike treatments involving acetaldehyde addition only, treatments with low pH only did not increase polymeric pigment concentration to the same extent, despite being as effective in preserving red color at 50 and 60 °C (Table 4). This suggests that the mechanism for color stabilization due to low pH was different from acetaldehyde color stabilization. Anthocyanin conversion at low pH could be attributable to direct condensation with flavan-3-ols and tannins, whereas acetaldehyde treatment could permit both direct condensation and acetaldehyde-mediated condensation reactions. At 70 °C, anthocyanin hydrolysis at low pH appeared to be the cause of significant anthocyanin and red color loss in low pH only treatments, compared to the control (Table 4).

Malvidin-3-*O*-glucoside was present at a much lower concentration in Rubired concentrate, approximating 5% that of malvidin-3,5-*O*-diglucoside. The concentration of malvidin-3-*O*-glucoside showed different trends to the diglucoside at 50 and 60 °C. Low pH increased malvidin-3,5-*O*-diglucoside reactivity while decreasing reactivity of the less stable malvidin-3-*O*-glucoside. All treatments at low pH had significantly higher malvidin-3-*O*-glucoside concentrations compared to the control and seed tannin treatment, with the acetaldehyde treatment giving the lowest concentration, likely due to pyranoanthocyanin-forming reactions (Table 4).

**Figure 2:**
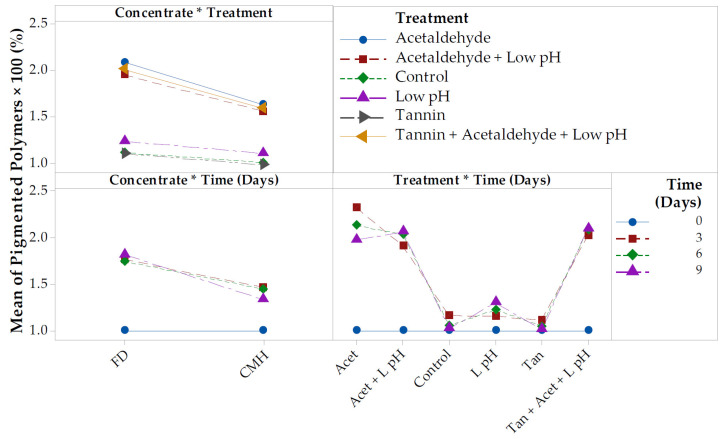
Fitted means for interaction plot for pigmented polymers in Rubired concentrate from conventional must heating (CMH) vs. flash détente (FD) heated at 50 °C; α = 0.05; *n* = 2. All data normalized to 68 °Brix and to the initial concentration to enable calculation of percentage change.

The increased pigmented polymer concentration in FD concentrate (~160%) compared to CMH concentrate (~130%) after heat treatment (Table 2) could be attributed to FD concentrate having a higher proportion of color in anthocyanin form. The difference in pigmented polymer increase between the two concentrates cannot be explained by malvidin-3,5-*O*-diglucoside and malvidin-3-*O*-glucoside loss, as these were fairly similar. However, conversion of other major anthocyanins in Rubired such as 3,5-diglucosides of peonidin and petunidin, and delphinidin mono and diglucosides [39,40] may possibly explain the differences in pigmented polymer formation. Indeed, the concentrate type by treatment interaction for pigmented polymer formation was statistically significant (Table 2). All treatments involving acetaldehyde addition had about double the pigmented polymer concentration of the control, seed tannin, and low pH treatments. In addition, acetaldehyde treatments had greater increases in pigmented polymer concentrations in FD concentrate compared to CMH concentrate (Figure 2). The FD concentrate initially had higher anthocyanin and lower pigmented polymer concentrations compared to CMH concentrate, and the lower pigmented polymer formation in CMH concentrate may point to equilibrium effects limiting anthocyanin conversion into more stable pigments.

### 3.4. Brown Color Evolution

Brown color formation during accelerated aging was greatly influenced by storage temperature (Table 1), acetaldehyde treatment, and pH, whereas seed tannin and concentrate type had no effect (Table 2 and Table 3). Brown color units exhibited different trends depending on storage temperature. After nine days of accelerated aging, control CMH and FD concentrates had decreased in brown color at 50 °C by 17–23%, whereas they had increased by 75–77% at 70 °C (Table 1).

All treatments involving acetaldehyde addition significantly increased brown color units in both CMH and FD concentrates after nine days of heating at 50 or 60 °C, but not at 70 °C, where only treatment with acetaldehyde alone gave a similar effect (Table 3). This was consistent with findings in model wine showing that acetaldehyde reacted with flavan-3-ols, which are known to be present in grape concentrate, to form yellowish flavanol-ethyl-flavanol adducts [41]. In addition to brown color arising from such adducts, anthocyanins can react with acetaldehyde to form pyranoanthocyanins such as vitisins [37] as indicated earlier, which are red pigments with a higher proportion of brown color compared to anthocyanins [38]. At 50 or 60 °C, the observed increase in brown color of low pH concentrate (Table 3) potentially arose from the reaction of flavan-3-ols with glyoxylic acid arising from oxidation of tartaric acid to form a carboxymethine bridge-linked adduct that can undergo further reaction to form a yellowish xanthylium compound [42]. This resulted in intermediate brown color units compared to seed tannin treatment and control that had the lowest brown color units after nine days of accelerated aging.

**Figure 3:**
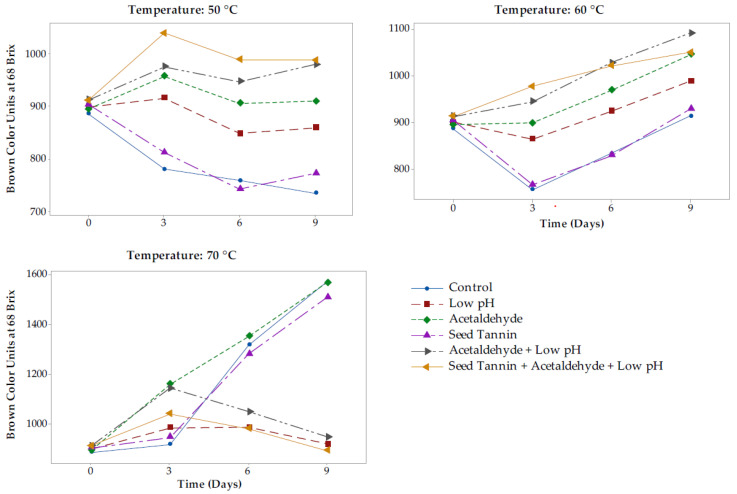
Effect of seed tannin, low pH, and acetaldehyde on brown color evolution in Rubired concentrate from conventional must heating (CMH) at different temperatures. All data normalized to 68 °Brix.

Compared to the initial color, brown color units increased in all treatments involving acetaldehyde addition and low pH but decreased in control and tannin treated concentrates after heating at 50 and 60 °C (Figure 3). At 70 °C, brown color units in concentrates from all low pH treatments initially increased, but then started to decrease after three days of accelerated aging, resulting in significantly lower brown coloration (Table 3). It is postulated that brown color loss in all low pH treatments heated at 70 °C was due to acid hydrolysis of brown colored flavanol-ethyl-flavanol adducts that have previously been reported to be susceptible to acid hydrolysis [43].

The trend for the brown index (ratio of brown to red color) was very consistent for the two types of concentrates at each of the temperatures studied (Table 3). Seed tannin treatments and controls gave the highest brown index, followed by acetaldehyde addition only, with low pH treatments giving the lowest ratios. A significantly greater decrease in red color (compared to brown color) had the effect of increasing the brown index in tannin treatments and controls during accelerated aging.

Low pH treatments with acetaldehyde addition had a significantly lower brown index, despite having significantly higher brown color units at 50 and 60 °C (Table 3). This was due to the increase in brown color units in combined low pH and acetaldehyde treatments being counteracted by a greater increase in red color units, due to the increased formation of the more stable pyranoanthocyanins and pigmented polymers (Table 4). On the other hand, the lower brown index in all low pH treatments at 70 °C was mainly associated with the loss of brown color.

Different browning mechanisms appear to be at play, depending on temperature and pH. Control and seed tannin treatments, which were at a normal Rubired concentrate pH of 4.0, developed less brown color after heating at 50 and 60 °C compared to other treatments involving acetaldehyde addition or low pH. This suggests that brown color formation at these temperatures was driven by acid-catalyzed pyranoanthocyanin and xanthylium compound formation and not by caramelization or the Maillard reaction, since the rate of brown color formation for the two latter reactions increases with pH [44,45,46]. The two-fold increases in brown color observed in concentrates at their original pH and after heating at 70 °C may be largely due to caramelization of sugars at the higher temperature, whereas no significant caramelization appears to take place below 55 °C [47].

In addition to the brown pigments formed via the Maillard reaction and caramelization (i.e., melanoidins), furfural and 5-hydroxymethylfurfural (5-MHF) derived from these reactions may have further contributed to browning due to their reaction with the flavanols present in the concentrate, forming yellow-orange xanthylium salts [48].

The complexity of grape juice means that some inferences have to be made, and the broader food chemistry knowledge needs to be drawn upon to rationalize the outcomes. To further understand the extraction process and color stabilization phenomena, work was undertaken to assess the impact of treatments on other concentrate constituents that may potentially affect color, quality, and product safety. This involved quantifying 5-HMF, caftaric acid, grape reaction product (GRP), proanthocyanidins, gallic acid, and quercetin glycosides during aging.

### 3.5. Impact of Treatments on Concentrate Quality Indicators

#### 3.5.1. 5-Hydroxymethylfurfural (5-HMF) Formation

5-HMF concentrations increased to a significantly greater extent in CMH concentrate compared to FD concentrate during accelerated aging (Table 2). It was hypothesized that the longer heat exposure during CMH concentrate production generated more 5-HMF precursors, leading to a much faster rate of 5-HMF formation during aging (Figure S2). Low pH significantly increased 5-HMF formation at all temperatures studied, whether from caramelization or the Maillard reaction, in agreement with previous research [49]. In contrast, acetaldehyde and seed tannin had no effect on 5-HMF formation (Table S1).

All low pH concentrates had on average approximately two times the concentration of 5-HMF than concentrates with unadjusted pH, following accelerated aging at 50, 60, and 70 °C (Figure S3). For all low pH treatments, 5-HMF formation was positively correlated with brown color at 50 and 60 °C, but negatively correlated at 70 °C, with the latter being consistent with previous research [50].

Although the combined treatment involving low pH and acetaldehyde addition was most effective at preserving red color, this also resulted in significantly higher 5-HMF concentrations irrespective of temperature, and at 50 and 60 °C, higher brown color units as outlined above in Section 3.4. High brown color units are detrimental to concentrate quality, and higher 5-HMF concentrations are undesirable because of potential in vivo conversion to 5-sulfoxymethylfurfural, which is a known genotoxin [51]. Additionally, some countries (e.g., in the European Union) impose limits on the allowable concentration of 5-HMF in grape juice concentrate. Formation of 5-HMF will likely be a concern where concentrate is stored for extended periods of time before use. Further research is therefore needed to determine methods for stabilizing red grape color without increasing the brown color or 5-HMF concentration of concentrate.

#### 3.5.2. *trans*-Caftaric Acid and 2-*S*-Glutathionyl Caftaric Acid (Grape Reaction Product)

Caftaric acid concentrations were monitored to compare the oxidative state of the two concentrates, as well as to determine if enzymatic oxidation played a role in brown color formation during aging. FD concentrate initially contained ~four-fold higher caftaric acid concentrations than CMH-derived concentrate, suggesting that the higher temperature treatment for a shorter duration of time used to produce FD resulted in less oxidation than the lower temperature treatment for a longer duration of time employed during CMH production. The caftaric acid levels in the control concentrate generally showed an increasing trend during accelerated aging, suggesting that enzymatic oxidation by polyphenol oxidase did not play a role in brown color formation, as previously reported [52], due to heat inactivation of oxidative enzymes [53].

Seed tannin and acetaldehyde treatments did not significantly impact caftaric acid concentrations (Table 4), while the low pH treatments gave lower caftaric acid concentrations (compared to controls) after nine days of accelerated aging, possibly due to acid hydrolysis to give caffeic and tartaric acids [54]. GRP concentrations were not affected by any of the treatments after heating at 50 and 60 °C, but the combination of low pH and high temperature decreased the GRP concentration at 70 °C (Table 4), likely due to acid hydrolysis of 2-*S*-glutathionyl caftaric acid into 2-*S*-glutathionyl caffeic acid [54].

The caftaric acid trend for all low pH treatments at 70 °C (Figure S4) was similar to that observed for brown color. Although the caftaric acid concentration initially increased (i.e., up until day three), the concentration dropped to its initial concentration by day nine. This suggested a similar reaction mechanism, i.e., acid hydrolysis, may have contributed to the decrease in both brown color and caftaric acid concentrations.

#### 3.5.3. Proanthocyanidin, Gallic Acid, and Quercetin Glycosides

Proanthocyanidin and gallic acid concentrations increased during accelerated aging (Figures S5, S6), potentially due to polymerization of monomeric and oligomeric flavan-3-ols [55] and hydrolysis of gallate esters [56], respectively. There were no significant differences between the proanthocyanidin concentrations of CMH and FD concentrates, but there was a general increase with increasing temperature for all treatments (Figure S5). Low pH and acetaldehyde treatments, either individually or in combination, gave higher proanthocyanidin concentrations after nine days of heating at 50 or 60 °C, while seed tannin additions had less impact (Table 4). Proanthocyanidin concentrations in the low pH treatments were lower than the control after nine days of heating at 70 °C, possibly due to increased acid hydrolysis at the higher temperature [57]. Seed tannin treatments gave higher or similar gallic acid concentrations compared to the control (Table 4), whereas low pH treatments gave lower gallic acid concentrations at 50 and 60 °C. At 70 °C, gallic acid concentrations increased and plateaued in all concentrates after three days of heating, decreasing thereafter possibly due to thermal degradation to yield no significant difference amongst treatments (Table 4, Figure S6). The concentration of quercetin glycosides, a co-pigmentation cofactor with the potential to influence red color intensity and stability [58,59], was monitored during aging. Quercetin glycoside concentrations decreased during accelerated aging of all treatments, possibly due to acid hydrolysis (Table 2).

#### 3.5.4. Sediment Formation

**Figure 4:**
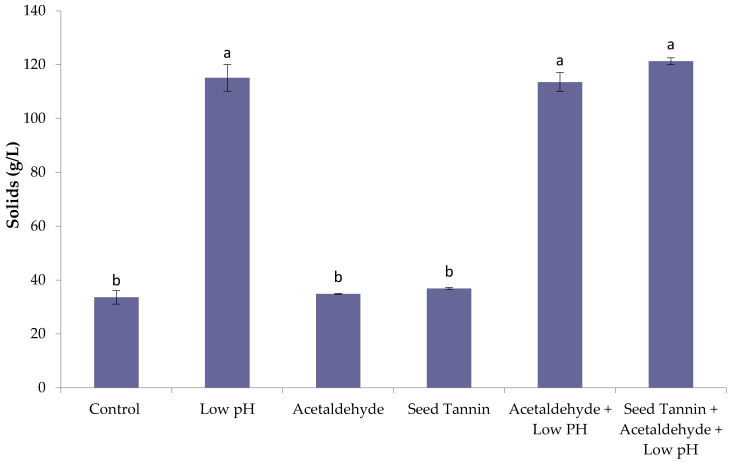
Precipitate formation in Rubired concentrate derived from conventional must heating (CMH) after 12 days of accelerated aging at 70 °C. Values are means of two replicates ± standard deviation; bars sharing the same letter are not significantly different; α = 0.05; *n* = 2; Tukey pairwise comparisons.

Heating the concentrates to accelerate the aging process resulted in sediment formation in all concentrates. The quantity and composition of sediment was therefore measured to determine if precipitation accounted for some of the losses in color and phenolic compounds that were observed during heat treatment. The sediments were insoluble in water, but soluble in 30% aqueous ethanol. Significantly higher sediment formation was observed at 70 °C, compared to 50 and 60 °C, with treatments involving low pH giving three times the quantity of solids as that for control, seed tannin, and acetaldehyde treatments (Figure 4).

There was no significant difference in sediment red and brown coloration amongst treatments (Table S2), suggesting precipitation did not account for the color losses observed for concentrates (Table 3). The brown color of sediments was positively correlated to sediment mass (Pearson’s r = 0.79). Together these results suggest that the brown color of sediments was due to adsorption to solid particles and not the result of brown color precipitation driving sediment formation.

Phenolic compounds in sediments represented less than 10% of the amount determined for the concentrates. Sediment collected from 50 mL samples of concentrate after 12 days of heating at 70 °C had relatively small amounts of total tannin (27–65 mg), proanthocyanidins (7–18 mg), and pigmented polymers (1 mg), with the lowest amounts observed for control concentrate, and the highest amounts for treatments involving both low pH and acetaldehyde addition (Table S2). Concentrations of malvidin-3-*O*-glucoside, catechin, epicatechin, gallic acid, caftaric acid, and quercetin glycosides were below the detection limits in all sediments. The higher amounts of phenolic compounds from all low pH treatments that had higher red color stability suggested that phenolic precipitation had no effect on color retention in concentrates. The presence of tannin in precipitates was consistent with findings reported by other researchers [55], who showed acetaldehyde-mediated crosslinking of catechin increased the mean degree of polymerization (MDP) of catechin-catechin polymers, leading to some tannin precipitation. Similar to findings from the current study, the precipitates were reported to be ethanol soluble, but water insoluble [50].

The concentration of 5-HMF in sediment was positively correlated (Pearson’s r = 0.99) to sediment mass, and ranged from 12–81 mg (Table S2), with seed tannin and acetaldehyde treatments having 5-HMF masses that were ~1.5 times that of the control, whereas all low pH treatments had amounts that were ~6.4 times greater, on average. As a result, sediments from low pH concentrates had around twice the 5-HMF concentration (i.e., g of 5-HMF/g of sediment) of control sediment, despite having ~3 times the mass of sediment than the control. It is therefore likely that sediment formation was linked to caramelization and the Maillard reaction, whose mechanisms follow different pathways depending on pH and temperature [49].

### 3.6. Color Degradation Kinetics

Reaction orders, rate constants, calculated activation energies, half-lives, and Q10 values were determined during accelerating aging (Table 5). Reactions were first order for red color, malvidin-3,5-*O*-diglucoside and malvidin-3-*O*-glucoside degradation, and zero order for browning index and violet color formation. The reaction order determined for anthocyanins was consistent with the reaction order that has been reported for malvidin-3-*O*-glucoside in model systems [59]. Proanthocyanidin formation followed zero order kinetics while quercetin glycoside degradation followed first order reaction kinetics. Additionally, red color in CMH concentrate was more stable at 50 °C, as shown by a half-life of 233.9 h and activation energy of 65.2 kJ/mol compared with 203.3 h and 59.2 kJ/mol for FD concentrate (Table 5).

The reaction orders for brown color and pigmented polymer formation were indeterminate, as differing trends were observed at the different temperatures studied. Brown color units decreased at 50 and 60 °C but showed an increasing trend at 70 °C. On the other hand, pigmented polymer concentration was unchanged at 50 and 60 °C, but increased at 70 °C. As a result, pigmented polymer formation did not conform to any simple reaction order. Brown index followed zero order kinetics. Other researchers [47] have also reported zero order kinetics for brown color formation in pekmez (a molasses-like syrup made with grape juice) stored at 55, 65, and 75 °C for 10 days at pH 4.0.

## 4. Conclusions

This research showed that FD concentrate had lower color stability compared to CMH concentrate, making it less desirable for applications where high color stability is a key requirement. On the other hand, FD concentrate had significantly lower 5-HMF formation, which might make it more suitable in low pH, long shelf-life food and beverage applications where 5-HMF formation may be a concern. The study also demonstrated beneficial red and violet color stabilization effects due to treatments involving low pH and acetaldehyde addition to Rubired concentrate, with an additive effect observed, which appeared to follow different mechanisms when these treatments were combined. However, the downside to low pH and acetaldehyde addition was increased brown color, with the former also increasing 5-HMF and sediment formation. The net effect to color quality was positive, because these treatments decreased the ratio of brown to red color. In contrast, seed tannin had no effect on red or violet color stability or brown color formation, regardless of the temperature used to accelerate aging.

It was evident from this work that the ideal treatment conditions for Rubired color stabilization are temperature dependent. When deciding on a treatment, consideration should be given to the level of heating that juice and concentrate will be subjected to during production, or as an ingredient in a food or beverage product during subsequent processing. Consideration should also be given to the acidity of foods and beverages, and to the temperature and duration of storage of the concentrate, as well as that of foods and beverages after colorant addition.

Whereas some mechanisms have been proposed for red color loss to explain how low pH and acetaldehyde might increase red color stability and brown color formation, the mechanism of brown color loss is not well understood. Research is therefore needed with the aim of developing solutions for remediating browning in concentrates, which is generally considered detrimental to quality. This is in addition to studies that provide red color stabilization without increasing brown coloration in juice concentrates to determine if findings from this research can be implemented under normal juice and concentrate production and storage conditions. Finally, research will be required to understand consumer acceptability of concentrates and juices with a higher violet to red color ratio due to acetaldehyde treatment.

## Figures and Tables

**Table 1:** Percentage change in red, violet, and brown color of concentrate derived from commercial must heating (CMH) vs. flash détente (FD) after nine days of accelerated aging (at 50, 60, and 70 °C).

Concentrate	Accelerated Aging Temperature								
	50 °C			60 °C			70 °C		
	Red	Violet	Brown	Red	Violet	Brown	Red	Violet	Brown
CMH	−47.3	−11.0	−17.0	−64.3	−21.9	−3.0	−69.4	−41.2	77.4
FD	−52.1	−10.7	−22.9	−68.8	−24.2	6.9	−72.4	−39.4	74.6

Values are means from two replicates.

**Table 2:** Change in chemical composition relative to initial values of concentrate derived from commercial must heating (CMH) vs. flash détente (FD) after nine days of accelerated aging (at 50 °C) and *p* values from repeated measures ANOVA.

	Means		*p* Values							Model Adjusted R^2^
	CMH	FD	Concentrate Type	Treatment	Time (days)	Concentrate Type × Treatment	Concentrate Type × Time	Treatment × Time	Concentrate Type × Treatment × Time	
Red color (%)	82.0	79.7	0.0005	0.0005	0.0005	0.201	0.007	0.0005	0.894	98.80%
Brown color (%)	98.5	99.0	0.242	0.0005	0.0005	0.0005	0.140	0.0005	0.0005	97.21%
Violet color (%)	110.1	114.1	0.0005	0.0005	0.0005	0.0005	0.0005	0.0005	0.0005	98.79%
Brown index	0.56	0.49	0.0005	0.0005	0.0005	0.005	0.050	0.0005	0.417	98.00%
Violet index	0.46	0.43	0.0005	0.0005	0.0005	0.001	0.034	0.0005	0.500	98.72%
5-Hydroxymethylfurfural (%)	9300	1900	0.0005	0.0005	0.0005	0.0005	0.0005	0.001	0.062	92.09%
Malvidin-3,5-*O*-diglucoside (%)	68.2	69.6	0.043	0.0005	0.0005	0.940	0.123	0.001	0.806	98.39%
Malvidin-3-*O*-glucoside (%)	52.3	53.0	0.398	0.0005	0.0005	0.961	0.308	0.0005	–	99.04%
Pigmented polymers (%)	131.2	158.2	0.0005	0.0005	0.0005	0.0005	0.0005	0.0005	0.0005	99.36%
Caftaric acid (%)	116.0	109.8	0.0005	0.0005	0.0005	0.018	0.0005	0.007	0.726	93.26%
Epicatechin (%)	38.9	39.2	0.870	0.245	0.0005	0.219	–	–	–	98.48%
Gallic acid (%)	132.9	150.5	0.0005	0.0005	0.0005	0.0005	0.0005	0.0005	0.0005	97.43%
Grape reaction product (%)	88.3	89.6	0.002	0.001	0.0005	0.350	0.132	0.058	0.998	96.92%
Proanthocyanidins (%)	126.8	128.1	0.086	0.0005	0.0005	0.0005	0.0005	0.0005	0.002	98.30%
Quercetin glycosides (%)	87.0	89.2	0.0005	0.0005	0.0005	0.527	0.005	0.0005	0.855	97.14%

Values are means from control and treated samples for each concentrate type. All data normalized to 68 °Brix to enable calculation of percentage change; α = 0.05; *n* = 2.

**Table 3:** Comparison of red, violet, and brown color (normalized to 68 °Brix), and brown index of concentrate derived from commercial must heating (CMH) and flash détente (FD) after treatment with different additives and nine days of accelerated aging at different temperatures.

Treatment	Temperature	CMH				FD			
		Red Color	Violet Color	Brown Color	Brown Index	Red Color	Violet Color	Brown Color	Brown Index
Acetaldehyde + low pH	50 °C	1534 a	865 a	981 a	0.64 c	1685 a	920 a	959 ab	0.57 c
Seed tannin + acetaldehyde + low pH		1531 a	877 a	988 a	0.65 bc	1711 a	937 a	1007 a	0.59 bc
Low pH		1352 b	711 c	860 bc	0.64 c	1445 b	692 b	794 c	0.55 c
Acetaldehyde		1255 c	784 b	911 ab	0.73 ab	1394 b	877 a	934 b	0.67 a
Control		1017 d	569 d	735 d	0.73 ab	1039 c	558 c	655 d	0.63 ab
Seed tannin		1017 d	567 d	773 cd	0.79 a	1063 c	568 c	703 d	0.66 a
Acetaldehyde + low pH	60 °C	1068 a	749 a	1092 a	1.03 d	1136 a	764 a	1053 a	0.93 c
Seed tannin + acetaldehyde + low pH		1026 b	711 b	1051 a	1.03 d	1123 a	752 a	1029 ab	0.92 c
Low pH		895 c	644 c	989 b	1.10 c	942 b	651 b	946 b	1.01 b
Acetaldehyde		905 c	657 c	1046 ab	1.16 b	981 b	692 ab	982 ab	1.00 b
Control		688 d	499 d	913 c	1.33 a	677 c	474 c	790 c	1.17 a
Seed tannin		697 d	502 d	928 c	1.33 a	689 c	483 c	793 c	1.15 a
Acetaldehyde + low pH	70 °C	658 a	421 a	1569 a	2.38 b	698 a	446 a	1497 a	2.15 b
Seed tannin + acetaldehyde + low pH		591 b	376 b	1572 a	2.66 a	599 b	379 b	1482 ab	2.470 a
Low pH		577 b	368 b	1510 b	2.60 a	595 b	379 b	1428 b	2.40 a
Acetaldehyde		452 c	304 c	947 c	2.10 c	523 c	340 c	1007 c	1.93 c
Control		418 cd	277 c	893 d	2.14 c	498 cd	325 c	953 c	1.92 c
Seed tannin		404 d	276 c	919 cd	2.28 b	457 d	308 c	970 c	2.12 b

Values are means from two replicates. Means followed by different letters (within columns, at each temperature) are significantly different; α = 0.05; *n* = 2; Tukey’s pairwise comparisons.

**Table 4:** Comparison of phenolic compounds (mg/L, normalized to 68 °Brix) in concentrate derived from flash détente (FD) after nine days of accelerated aging at different temperatures.

Treatment	Temperature	Malvidin-3,5-*O*-diglucoside	Pigmented Polymers	Malvidin-3-*O*-glucoside	Proanthocyanidins	Gallic Acid	Quercetin Glycosides	*trans*-Caftaric Acid	GRP
Control	50 °C	2098 ab	104 b	79 c	1293 b	37 a	154 abc	106 a	44 a
Seed tannin		2142 a	109 b	90 bc	1418 b	41 a	157 ab	108 a	45 a
Acetaldehyde		1801 cd	243 a	29 d	2072 a	36 a	138 d	108 a	43 a
Low pH		1839 bc	139 b	241 a	1541 b	22 b	160 a	100 b	46 a
Acetaldehyde + low pH		1539 de	246 a	135 b	1970 a	21 b	150 bc	100 b	46 a
Seed Tannin + acetaldehyde + low pH		1436 e	253 a	119 bc	2197 a	27 b	145 cd	99 b	45 a
Control	60 °C	431 a	99 d	42 c	1883 e	43 b	83 abc	97 a	29 a
Seed tannin		439 a	101 d	43 c	2030 e	49 a	84 ab	96 a	29 a
Acetaldehyde		377 b	169 b	18 d	2479 d	41 b	73 d	95 a	29 a
Low pH		157 c	149 c	139 a	2965 c	31 cd	88 a	81 b	30 a
Acetaldehyde + low pH		127 c	211 a	72 b	3250 b	28 d	81 bc	81 b	30 a
Seed tannin + acetaldehyde + low pH		124 c	220 a	67 b	3589 a	34 c	79 c	80 b	30 a
Control	70 °C	51 a	148 c	nd	4605 b	36 a	16 a	79 b	28 a
Seed tannin		48 ab	139 d	nd	4637 b	42 a	15 a	82 a	28 a
Acetaldehyde		45 b	183 a	nd	5092 a	36 a	15 a	83 a	28 a
Low pH		30 c	137 d	nd	4462 b	37 a	18 a	58 c	22 b
Acetaldehyde + low pH		29 c	177 a	nd	4910 a	41 a	17 a	59 c	24 ab
Seed Tannin + acetaldehyde + low pH		29 c	164 b	nd	4525 b	44 a	17 a	59 c	22 b

Values are means from two replicates. Means followed by different letters (within columns, at each temperature) are significantly different; α = 0.05; *n* = 2; Tukey’s pairwise comparisons. All data normalized to 68 °Brix. nd, not detected.

**Table 5:** Kinetic parameter data for color, phenolic compounds, and 5-hydroxymethylfurfural (5-HMF) for concentrates derived from conventional must heating (CMH) and flash détente (FD) concentrates after nine days of accelerated aging at 50 °C.

	Concentrate Type	Reaction Order	Rate Constant (k) at 50 °C	Half Life (h) at 50 °C	Activation Energy (kJ/mol)	Q10
Red color	CMH	1	4.9 × 10^−5^	233.9	65.2	1.61
	FD		5.7 × 10^−5^	203.3	59.2	1.58
Brown index	CMH	0	2.0 × 10^−5^	187.5	224.7	3.26
	FD		1.9 ×10^−5^	175.5	229.3	3.25
Violet color	CMH	0	5.4 × 10^−3^	992.4	141.3	2.02
	FD		5.1 × 10^−3^	1012.0	138.4	2.27
Malvidin-3,5-O-diglucoside	CMH	1	7.2 × 10^−5^	161.2	171.7	2.87
	FD		6.6 × 10^−5^	175.1	177.8	2.85
Malvidin-3-O-glucoside	CMH	1	1.8 × 10^−4^	65.4	-	2.93
	FD		1.8 × 10^−4^	63.6	-	2.66
Quercetin glycosides	CMH	1	2.6 × 10^−5^	437.1	213.3	2.70
	FD		2.2 × 10^−5^	531.2	233.5	3.17
Proanthocyanidins	CMH	0	2.5 × 10^−2^	421.8	240.3	2.86
	FD		1.8 × 10^−2^	509.3	291.9	3.61

## Supplementary Materials

The following are available online at https://www.mdpi.com/2304-8158/9/9/1270/s1, Figure S1. Flowchart of conventional must heating vs. flash détente processes for production of Rubired concentrate; Figure S2. Fitted means for interaction plot for 5-hydroxymethylfurfural (5-HMF) in Rubired concentrate from conventional must heating (CMH) vs. flash détente (FD), heated at 50 °C; α = 0.05; n = 2. All data normalized to 68 °Brix and to initial concentration to enable calculation of percentage change; Figure S3. Effect of seed tannin, low pH, and acetaldehyde on 5-hydroxymethylfurfural (5-HMF) formation in Rubired concentrate from conventional must heating (CMH) at different temperatures. All data normalized to 68 °Brix. Note the different y-axis scales; Figure S4. Effect of seed tannin, low pH, and acetaldehyde on caftaric acid concentration in Rubired concentrate from conventional must heating (CMH) at different temperatures. All data normalized to 68 °Brix; Figure S5. Change in proanthocyanidin concentration during accelerated aging of Rubired concentrate from conventional must heating (CMH) at different temperatures. Note the different y-axis scales; Figure S6. Change in gallic acid concentration during accelerated aging of Rubired concentrate from conventional must heating (CMH) at different temperatures. All data normalized to 68 °Brix. Note the different y-axis scales; Table S1. Comparison of 5-hydroxymethylfurfural (5-HMF) concentrations in Rubired concentrate from conventional must heating (CMH) vs. flash détente (FD), after nine days of accelerated aging at different temperatures; Table S2. Sediment composition of concentrate from conventional must heating after 12 days of accelerated aging at 70 °C.
