# Supplementary material for: Impact of Juice Extraction Method (Flash Détente vs. Conventional Must Heating) and Chemical Treatments on Color Stability of Rubired Juice Concentrates under Accelerated Aging Conditions
Source: Foods. 2020 Sep 10;9(9):1270. doi: 10.3390/foods9091270 (PMC7554961; doi:10.3390/foods9091270)
Supplement: Supplementary file 1 [file foods-09-01270-s001.pdf]

Supplementary Materials For

Impact of Juice Extraction Method (Flash Détente vs. Conventional Must Heating) and Chemical Treatments on Color Stability of Rubired Juice Concentrates under Accelerated Aging Conditions

Richard G. Ntuli [1,2], Ravi Ponangi [1], David W. Jeffery [2,*] and Kerry L. Wilkinson [2]

[1] E & J Gallo Winery, Process Technology, PO Box 1130, Modesto, CA, 95353, USA; richard.ntuli@ejgallo.com (R.G.N.); ravi.ponangi@ejgallo.com (R.P.)
[2] School of Agriculture, Food and Wine, The University of Adelaide, PMB 1, Glen Osmond, SA 5064, Australia; kerry.wilkinson@adelaide.edu.au (K.L.W.)
* Correspondence: email: david.jeffery@adelaide.edu.au (D.W.J.); Tel: +61-8-8313-6649

Table of Contents

Page

Figure S1. Flowchart of conventional must heating vs. flash détente processes for production of Rubired concentrate. S2

Figure S2. Fitted means for interaction plot for 5-hydroxymethylfurfural (5-HMF) in Rubired concentrate from conventional must heating (CMH) vs. flash détente (FD), heated at 50 °C; α = 0.05; n = 2. All data normalized to 68 °Brix and to initial concentration to enable calculation of percentage change. S3

Figure S3. Effect of seed tannin, low pH, and acetaldehyde on 5-hydroxymethylfurfural (5-HMF) formation in Rubired concentrate from conventional must heating (CMH) at different temperatures. All data normalized to 68 °Brix. Note the different y-axis scales. S4

Figure S4. Effect of seed tannin, low pH, and acetaldehyde on caftaric acid concentration in Rubired concentrate from conventional must heating (CMH) at different temperatures. All data normalized to 68 °Brix. S5

Figure S5. Change in proanthocyanidin concentration during accelerated aging of Rubired concentrate from conventional must heating (CMH) at different temperatures. All data normalized to 68 °Brix. Note the different y-axis scales. S6

Figure S6. Change in gallic acid concentration during accelerated aging of Rubired concentrate from conventional must heating (CMH) at different temperatures. All data normalized to 68 °Brix. Note the different y-axis scales. S7

Table S1. Comparison of 5-hydroxymethylfurfural (5-HMF) concentrations in Rubired concentrate from conventional must heating (CMH) vs. flash détente (FD), after nine days of accelerated aging at different temperatures. S8

Table S2. Sediment composition of concentrate from conventional must heating after 12 days of accelerated aging at 70 °C. S9

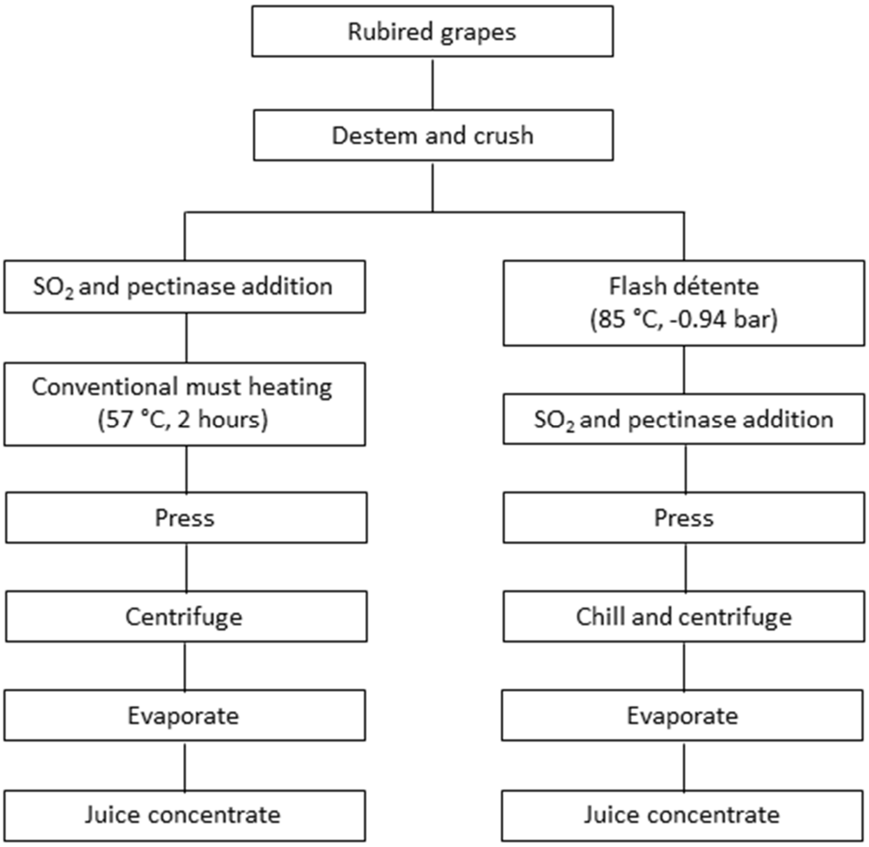

Figure S1. Flowchart of conventional must heating vs. flash détente processes for production of Rubired concentrate.

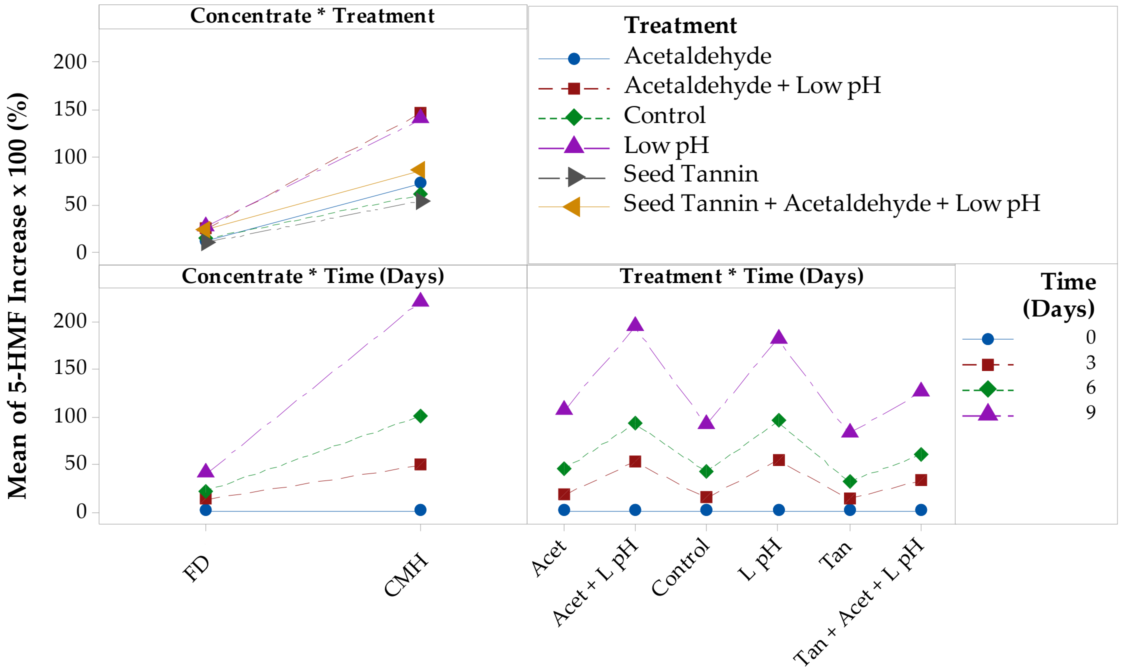

Figure S2. Fitted means for interaction plot for 5-hydroxymethylfurfural (5-HMF) in Rubired concentrate from conventional must heating (CMH) vs. flash détente (FD), heated at 50 °C; $\alpha = 0.05$; $n = 2$. All data normalized to 68 °Brix and to initial concentration to enable calculation of percentage change.

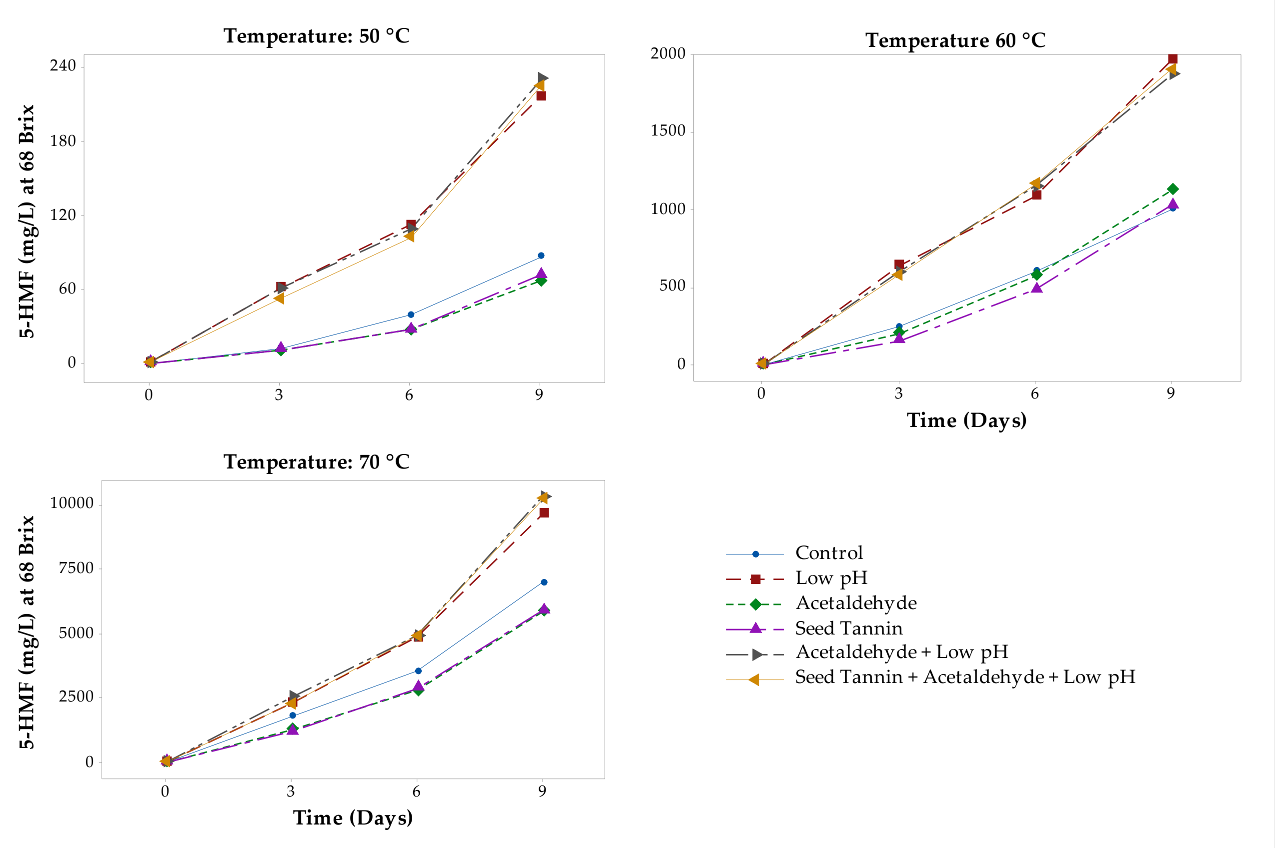

Figure S3. Effect of seed tannin, low pH, and acetaldehyde on 5-hydroxymethylfurfural (5-HMF) formation in Rubired concentrate from conventional must heating (CMH) at different temperatures. All data normalized to 68 °Brix. Note the different y-axis scales.

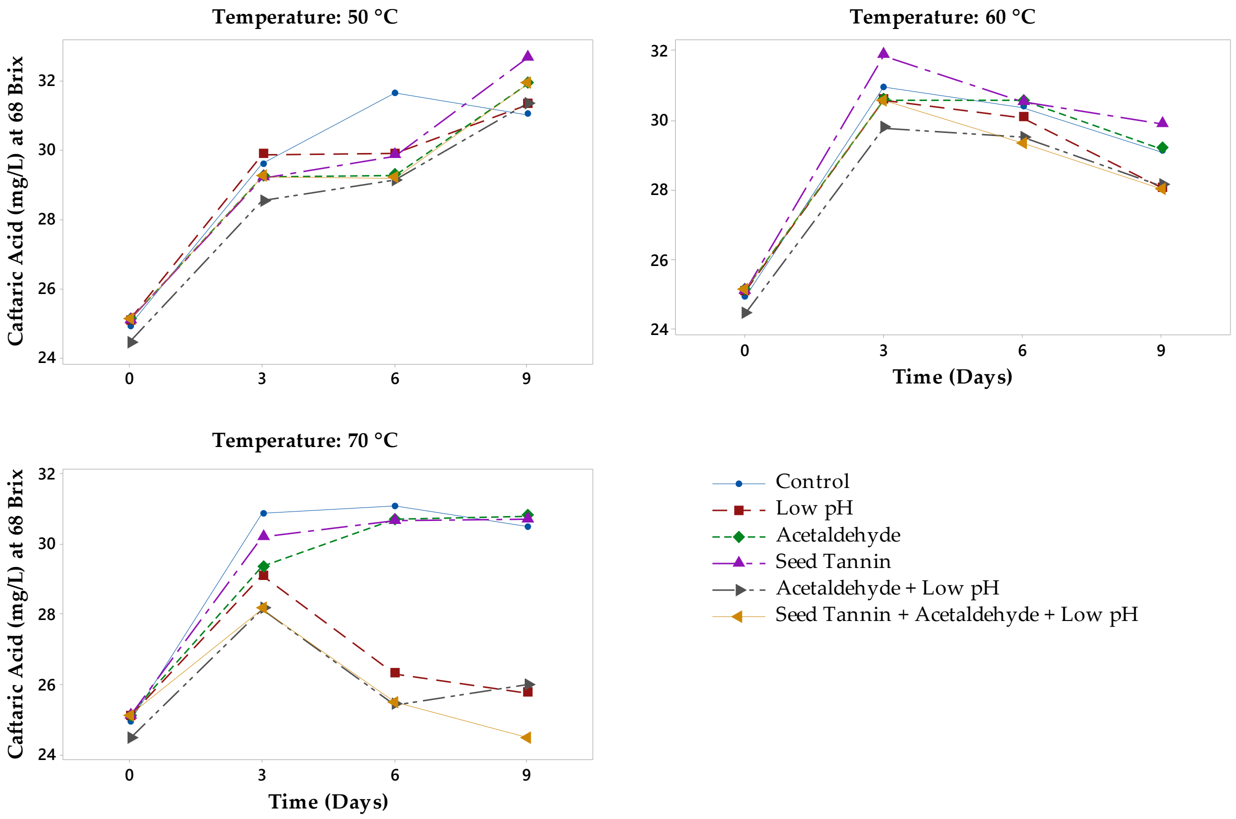

Figure S4. Effect of seed tannin, low pH, and acetaldehyde on caftaric acid concentration in Rubired concentrate from conventional must heating (CMH) at different temperatures. All data normalized to 68 °Brix.

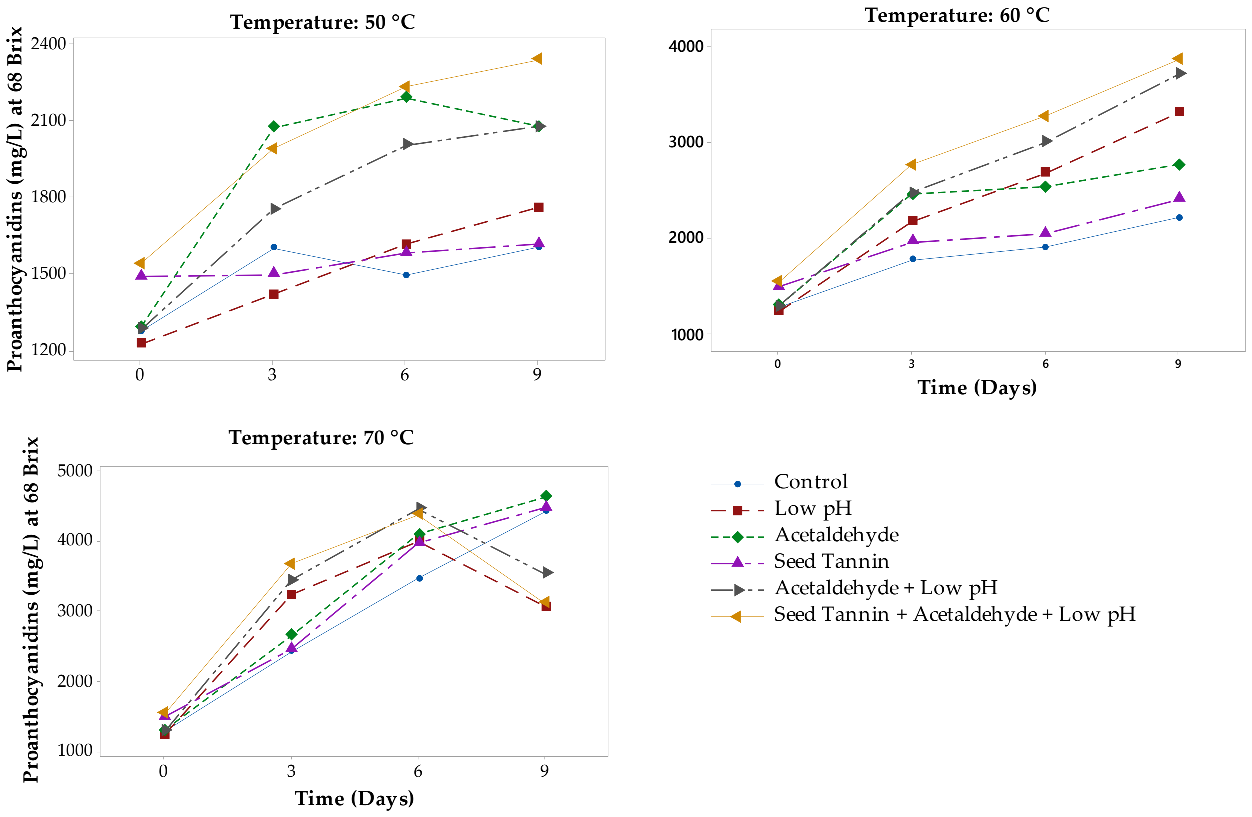

Figure S5. Change in proanthocyanidin concentration during accelerated aging of Rubired concentrate from conventional must heating (CMH) at different temperatures. All data normalized to 68 °Brix. Note the different y-axis scales.

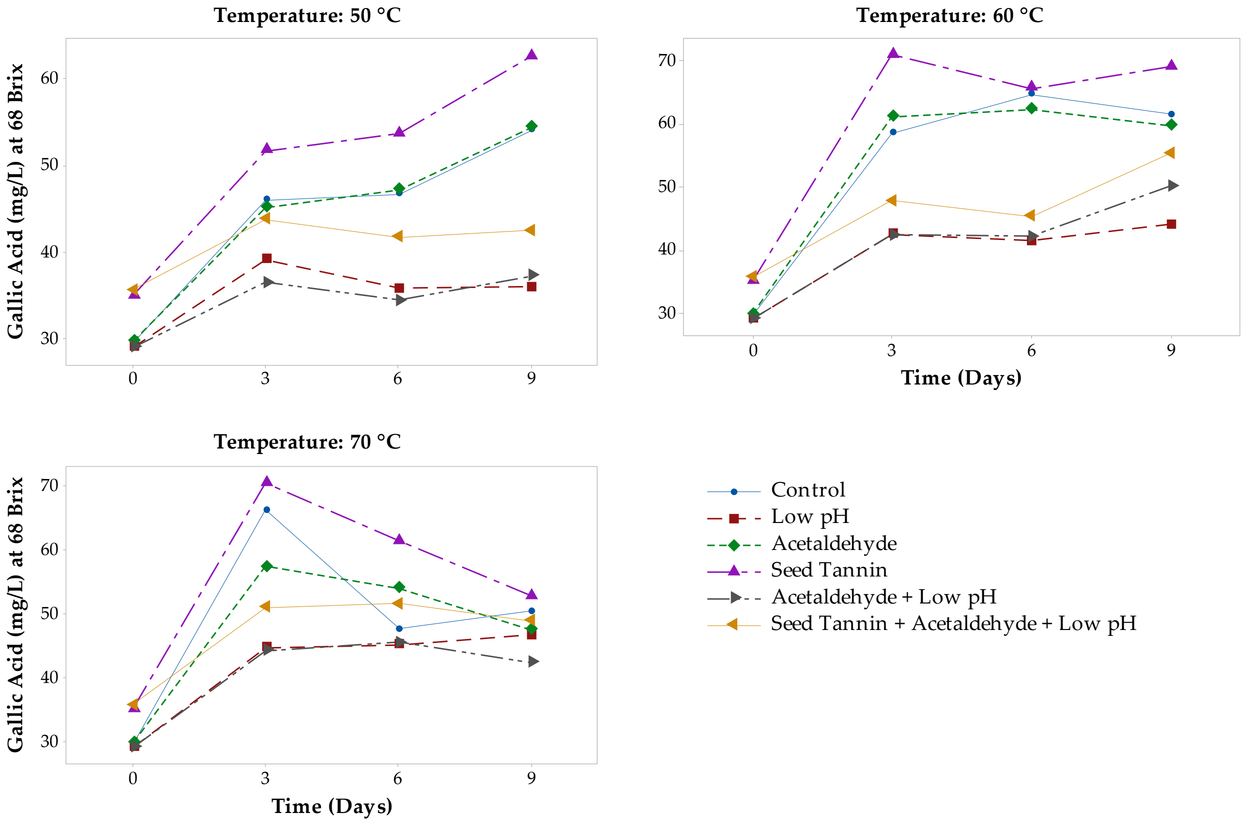

Figure S6. Change in gallic acid concentration during accelerated aging of Rubired concentrate from conventional must heating (CMH) at different temperatures. All data normalized to 68 °Brix. Note the different y-axis scales.

Table S1. Comparison of 5-hydroxymethylfurfural (5-HMF) concentrations in Rubired concentrate from conventional must heating (CMH) vs flash détente (FD), after nine days of accelerated aging at different temperatures.

Treatment	Temperature	CMH		FD	
		5-HMF (mg/L)	%RSD	5-HMF (mg/L)	%RSD
Seed Tannin + Acetaldehyde + Low pH	50 °C	225 a	9.5	176 a	12.3
Acetaldehyde + Low pH		230 a	19.5	186 a	20.9
Low pH		217 a	1.7	168 ab	13.4
Acetaldehyde		67 b	24.0	73 c	5.0
Seed Tannin		72 b	17.3	59 c	13.3
Control		87 b	23.2	87 bc	4.3
Seed Tannin + Acetaldehyde + Low pH	60 °C	1909 a	5.4	1580 a	2.5
Acetaldehyde + Low pH		1875 a	11.9	1769 a	4.8
Low pH		1969 a	1.3	1626 a	16.3
Acetaldehyde		1127 b	9.3	914 b	1.3
Seed Tannin		1030 b	3.8	858 b	6.3
Control		1005 b	7.8	874 b	0.1
Seed Tannin + Acetaldehyde + Low pH	70 °C	10234 a	6.9	8054 a	1.5
Acetaldehyde + Low pH		10317 a	3.8	7618 a	3.6
Low pH		9665 a	1.6	7056 ab	7.8
Acetaldehyde		5860 b	4.6	4694 c	4.5
Seed Tannin		5887 b	3.7	4676 c	0.8
Control		6975 b	6.2	6290 b	3.0

Data are means of two replicates (n = 2), normalized to 68 °Brix; %RSD = percentage relative standard deviation. Means followed by different letters (within columns, by temperature) are significantly different (α = 0.05, Tukey pairwise comparisons).

Table S2. Sediment composition of concentrate from conventional must heating after 12 days of accelerated aging at 70 °C.

Treatment	A420	A520	5-HMF (mg)	Proantho-cyanidins (mg)	Pigmented Polymers (mg)	Tannin (mg)	Sediment (g)
Control	3.12	1.14	11.91 b	7.00 b	0.86 b	26.6	3.36 b
Seed Tannin	6.66	2.64	20.17 b	12.38 ab	0.99 ab	52.9	3.69 b
Acetaldehyde	4.3	1.73	14.53 b	10.68 ab	0.97 ab	32.9	3.49 b
Low pH	6.91	2.79	72.02 a	16.68 ab	1.07 ab	62.1	11.51 a
Acetaldehyde + Low pH	7.41	3.21	80.86 a	17.61 a	1.16 a	64.8	11.35 a
Seed Tannin + Acetaldehyde + Low pH	7.51	3.25	75.3 a	17.86 a	1.16 a	64.6	12.13 a
p-value	ns	ns	0.0001	0.025	0.026	ns	0.0001
Pearson's Coefficient r with Sediment Mass	0.79	0.82	0.99	0.92	0.89	0.87	–

Data are means of two replicates ($n = 2$), normalized to 68 °Brix. Means followed by different letters (within columns) are significantly different ($\alpha = 0.05$, Tukey pairwise comparisons); ns = no significance.
